# Systematic review of atropine cost-effectiveness in childhood myopia management

**DOI:** 10.3389/fphar.2026.1836579

**Published:** 2026-10-16

**Authors:** Chee Fai Sui, Chin Hui Ng, Yee Chang Soh, Kah Seng Lee, Andi Hermansyah, Long Chiau Ming

**Affiliations:** 1 Faculty of Medical and Life Sciences, Sunway University, Sunway City, Malaysia; 2 Pharmacy Department, Hospital Raja Permaisuri Bainun, Ipoh, Malaysia; 3 PPD, Inc., Thermo Fisher Scientific, Kuala Lumpur, Malaysia; 4 Department of Pharmacy Practice, Faculty of Pharmacy, Universitas Airlangga, Surabaya, Indonesia; 5 Innovative Pharmacy Practice and Integrated Outcomes Research (INACORE), Faculty of Pharmacy Universitas Airlangga, Surabaya, Indonesia; 6 Research Center in Advancing Community Healthcare (REACH), Universitas Airlangga, Surabaya, Indonesia

**Keywords:** cost-utility analysis, economic evaluation, health technology assessment, Markov model, myopia control, pediatric ophthalmology, pharmacologic myopia intervention, vision disorders

## Abstract

**Background:**

Childhood myopia is a global public health concern, with rising prevalence and a significant economic burden due to complications like retinal detachment and macular degeneration. Low-dose atropine (LDA) range 0.01%–0.05% is a clinically effective intervention for slowing myopia progression. However, its economic value remains under-evaluated across diverse settings.

**Methods and Findings:**

We conducted a systematic review following PRISMA guidelines and registered in PROSPERO (CRD420251009680) to assess the cost-effectiveness of atropine for myopia management in children. Four model-based economic evaluations met the inclusion criteria, from Hong Kong, New Zealand, Australia/China and France/United Kingdom. Two studies used Markov cohort state-transition models while two employed a cohort decision-analytic model with probabilistic simulation of refractive outcomes. All four economic evaluations adopted a societal perspective; each applied a 3% annual discount rate to costs in their analyses and reported sensitivity analyses around discounting. Time horizons ranged from 5 years for incident myopia to lifetime horizons capturing myopia complications and vision impairment. Across economic models, LDA was consistently found to be cost-effective or cost-saving compared with standard care. Reported incremental cost-effectiveness ratios (ICERs) included approximately NZ$1,590 per quality-adjusted life year (QALY) gained in a New Zealand Markov cost–utility model, and USD 220–275 per diopter spherical equivalent reduction in a 5-year Hong Kong cost-effectiveness analysis. Evidence from high- and middle-income settings indicates that low-dose atropine is a cost-effective strategy, particularly when long-term progression to high myopia and its associated complications is considered. In European settings, low-dose atropine demonstrated a favorable cost profile compared with standard correction in children at higher risk of myopia progression, with consistent findings of cost-neutrality or cost savings when downstream costs of high myopia were included.

**Conclusion:**

Studies demonstrate favourable economic value using diverse metrics, including cost per diopter reduction, cost ratios, and cost–utility estimates, rather than uniformly reported ICERs. Greater economic benefits are observed over longer time horizons, largely driven by the avoidance of high myopia–related complications and associated costs.

**Systematic Review Registration:**

https://www.crd.york.ac.uk/PROSPERO/view/CRD420251009680, identifier PROSPERO CRD420251009680.

## Highlights

- Low-dose atropine is generally cost-effective for pediatric myopia control compared with traditional single-vision management in Hong Kong, China/Australia, New Zealand, and the UK/France.
- During the finite 2–10 years treatment period, the main short-term cost drivers are atropine acquisition and additional ophthalmology/optometry visits and monitoring, rather than the drops alone.
- In lifetime models, total costs are dominated by the refractive outcome achieved and the resulting risk of myopia-related complications and low-vision care, not by short-term atropine costs.
- Most existing economic evaluations use compounded, off-label low-dose atropine; the emergence of licensed pharmaceutical products (e.g., Eikance in New Zealand, Ryjunea in the Europe) means cost-effectiveness should be re-estimated using current prices and reimbursement pathways.
- Cost-effectiveness is highly context-dependent, varying with local drug pricing, prescribing and monitoring pathways (ophthalmologist vs. optometrist), and health-system financing, so ICERs from current models cannot be directly transferred to Malaysia or other ASEAN settings without local adaptation.

## 1 Introduction

Childhood myopia has reached epidemic proportions, with its global prevalence rising gradually from 24% in 1990 to 35% in 2023. Projections indicate the trend will continue, reaching 40% by 2040, approaching earlier estimates of 50% by mid-century (Holden et al., 2016; Liang et al., 2025). The global economic burden of myopia is substantial, with annual direct costs vision correction estimated at USD 328 billion (Chua and Foster, 2020). Additionally, high myopia (≥−6 diopters) poses significant lifelong risks, including irreversible vision loss due to complications such as macular degeneration, retinal detachment, and glaucoma. These severe cases contribute an extra USD 244 billion per year in direct healthcare expenses and productivity losses worldwide (Chua and Foster, 2020).

Pharmacological treatments for myopia, including low-dose atropine (0.01%), pirenzepine, apomorphine, and 7-methylxanthine (7-MX), offer diverse therapeutic options (Xu and Zhang, 2025). Among these, LDA has emerged as the frontline therapy, demonstrating the highest efficacy in clinical trials slowing myopia progression by 50%–60% with minimal side effects (Chia et al., 2016). Pirenzepine shows promise in animal models but is limited by poor corneal permeability, while apomorphine requires further clinical validation. 7-MX exhibits dose-dependent effects in slowing progression, though its efficacy needs broader population studies (Xu and Zhang, 2025). Thus, LDA remains the most evidence-based option, though research into alternatives continues to expand the field.

However, despite its well-established efficacy (Kaiti et al., 2022), adoption of LDA for myopia control remains inconsistent across countries, owing to differences in regulatory approval status, national clinical guideline recommendations, optimal dosing debates, long-term safety considerations, and critically unresolved questions about its economic value compared to alternatives like orthokeratology (VanderVeen et al., 2019) or multifocal lenses (Zhu et al., 2019).

Clinical studies confirm atropine’s efficacy across doses range from 0.01% to 1.0% (Chia et al., 2016; Ye et al., 2022; Zadnik et al., 2023). However, higher-dose atropine incurs cost and tolerability trade-offs due to side effect (Chia et al., 2016), while LDA though safer and cheaper, demonstrates strong efficacy in Asian populations (Zaabaar et al., 2024) but limited effectiveness in US studies (Repka et al., 2023). Consequently, there is a lack of real-world or trial-based cost-effectiveness studies especially in term of geographic diversity as well as standardized utilities-based outcomes.

To our best knowledge, this is the first systematic review of model-based economic evaluations on atropine for pediatric myopia. While atropine is a widely studied intervention for childhood myopia (Saxena et al., 2024; Zaabaar et al., 2024), its cost-effectiveness remains under-researched. To date, only one systematic review has included a cost-effectiveness analysis (CEA) of atropine, finding that 0.01% atropine combined with screening was cost-effective (Agyekum et al., 2023a). However, no dedicated systematic review has comprehensively synthesized economic evaluations of LDA for myopia control using model-based approaches. Therefore, this review aimed to systematically assess and compare model-based economic evaluations of LDA for childhood myopia control, focusing on cost-efficiency in relation to clinical outcomes such as delayed myopia progression.

## 2 Methods

### 2.1 Search strategy

This review was conducted in accordance with to the Preferred Reporting Items for Systematic Reviews and Meta-Analyses (PRISMA) framework (Page et al., 2021). The protocol was developed following the PRISMA-P 2015 checklist (Moher et al., 2015) and was registered in the International Prospective Register of Systematic Reviews (PROSPERO) under reference number CRD420251009680.

Figure 1 shows the PRISMA flow chart (Page et al., 2021) of the study retrieval and selection procedure.

**FIGURE 1:**
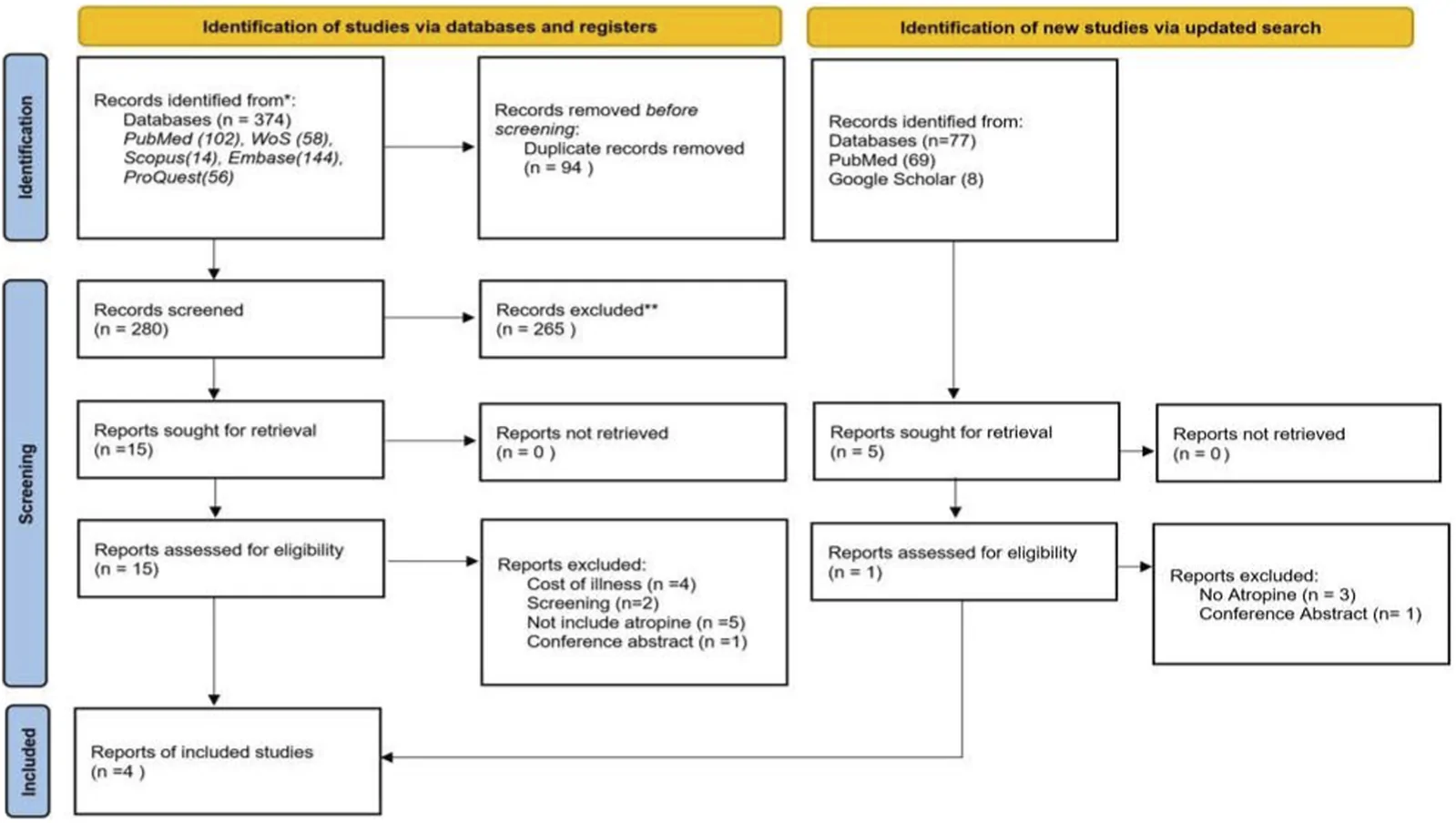
PRISMA flowchart.

A comprehensive literature search was conducted across multiple databases, including PubMed, Embase, the Cochrane Library, Web of Science (WOS), and ProQuest databases. The original search strategy was replicated from Agyekum et al. (2023a), whose cost-effectiveness analysis of myopia interventions included studies until June 2022. To ensure the review captured the most recent and relevant studies, we conducted a comparative literature search in PubMed up to 26 March 2025, using similar parameters to those employed by Agyekum et al. (2023a). Their original search identified 338 studies, whereas our updated search yielded 455 studies prior to applying filters specific to atropine. This increase reflects the expanding body of literature in the field. We supplement backward searching the reference lists of Agyekum et al. (2023a) and all included studies, adding any relevant publications that met our inclusion criteria.

Search terms utilized included “cost,” “myopia,” “nearsighted,” “shortsighted,” “cost-effectiveness,” and “economic evaluation.” These terms were selected and compared by the reviewers to ensure relevance and comprehensiveness.

Subsequently, additional searches were performed using the terms “medical intervention” and “atropine.” The results from these searches were compared between two researchers to ensure consistency and accuracy in the findings. The search was limited to studies published in English.

To ensure currency of the evidence base, a supplemental updated search was conducted on 15 May 2026 in PubMed and Google Scholar, restricted to publications from 2025 to 2026.

Search strategy is presented in Supplementary File S1 and PRISMA checklist is shown in Supplementary File S2.

### 2.2 Study selection

For the initial screen, titles and abstracts of studies identified by the search strategy were assessed for potential eligibility by two researchers independently. EndNote 20 and Rayyan software were used to organize the screening and review process. Papers included at the initial screen were retrieved, with the full text screened independently by two. Differences in opinion were resolved at each stage through discussion.

#### 2.2.1 Inclusion criteria

Studies were included if they met the following criteria:

1. Economic evaluation study—cost consequences, cost effectiveness, cost utility, cost benefit analysis
2. Atropine as an intervention
3. Reported economic outcomes such as cost per quality-adjusted life year (QALY) gained, incremental cost-effectiveness ratio (ICER), or net monetary benefit (NMB), lifetime cost estimate, or cost ratio (intervention vs. comparator)
4. Trial based or Analytical model of cost-effectiveness
5. Pediatric population as the primary target for atropine intervention (children aged ≤18 years at initiation of the modelled intervention)
6. Full test published in English language

Studies were excluded if they:

1. Did not provide sufficient economic data (cost data reported without comparator)
2. Not peer-reviewed articled
3. Assessed atropine for indications other than myopia management (e.g., amblyopia, cycloplegia for refraction)

### 2.3 Data extraction and management

Data extraction was performed independently by two researchers [CS and CN] using a standardized Excel form. Extracted data included study characteristics (author, year, country, sample size), intervention details (atropine concentration, duration), economic outcomes (costs, QALYs, ICERs), and methodological quality. Discrepancies were resolved through discussion or consultation with a third reviewer [L.C.M].

All reported costs were standardized to 2024 USD to enable cross-study comparison using the CCEMG–EPPI Centre Cost Converter v1.7 (Shemilt et al., 2010) and presented alongside original reported values in Table 1 for transparency. To facilitate cross-study comparison and reader comprehension, the intervention using atropine is uniformly described as low dose atropine (LDA) and the comparator as conventional optical correction (COC) throughout this review, consistent with terminology used in IMI clinical guidelines (Gifford et al., 2019).

**TABLE 1:** Summary of economic findings and convergent evidence synthesis across included studies (n = 4).

Field	Fricke et al. (2023) Australia / China	Hong et al. (2022) New Zealand	Agyekum et al. (2023b) Hong Kong / China	Lee et al. (2025) France / United Kingdom
Study design				
Economic design	CCA — Cost-Consequence Analysis	CUA — Cost-Utility Analysis	CEA — Cost-Effectiveness Analysis	CCA — Cost-Consequence Analysis
Model type	Probabilistic lifetime cohort cost model	Hybrid decision-tree + 5-state Markov model	3-state Markov model	Probabilistic lifetime cohort cost model (extended Fricke 2021 methodology)
Perspective	Societal	Societal	Societal	Societal
Cohort / Starting age	8-year-old child; −0.75 DS Australian (European) & Han Chinese	11-year-old children New Zealand population	10-year-old children Hypothetical cohort; −0.50 D low myopia	8-year-old child (male & female); −0.75 DS France & United Kingdom (4 devolved nations)
Time horizon	Lifetime (to life expectancy)	Lifetime (to age 80)	5 years	Lifetime (to life expectancy)
Atropine intervention	0.01–0.05% [AC] vs. [OC]	Photorefraction screening + [AC] 0.01% vs. [OC]	[AC] 0.05% and 0.01% vs. [OC]	[AC] 0.01–0.05% vs. [OC]
Discount rate	0%, 3%, 5% (primary: 3%)	3% (sensitivity: 6%)	3% (sensitivity: 0–6%)	0% (primary); 3% (sensitivity)
Currency (original / converted*)	USD 2020 / USD 2024*	NZD 2021 / USD 2024*	USD 2022 / USD 2024*	USD 2024
Costs				
Intervention (AC) total cost^1^ ^,^ ^2^	^1^Australia: USD 16878 ^1^China: USD 11526 Standardize 2024 ^1^Australia: USD 20008 ^1^China: USD 13664	NR	^2^HK-5 year Atropine 0.05%: USD 6251 Atropine 0.01%: USD 6193 Standardize 2024 Atropine 0.05%: USD 6628 Atropine 0.01%: USD 6566	^1^France : USD24,907(fast), USD23,354(slow)^1^UK : USD33,015(fast), USD29,605(slow)
Comparator (OC) total cost^1^ ^,^ ^2^	^1^Australia: USD 18381 ^1^China : USD 23636 Standardize 2024 ^1^Australia: USD 21790 ^1^China: USD 28020	NR	^2^HK: USD 4,372 Standardize 2024 USD 4635	^1^France faster: USD 32,492 | Slower: USD 22,606 ^1^UK faster: USD 48,170 | Slower: USD 29,664
Incremental cost (atropine vs. comparator)	Australia: cost-saving; [AC]= - USD 1503 Standardize 2024 = -USD 1782 China: cost-saving; [AC]= - USD 12110 Standardize 2024 = -USD 9972	NZD +17.70/person screened (Standardize USD 2024 = +13.22)	Atropine 0.05%: +USD 1,822 Atropine 0.01%: +USD 1,879 Standardize 2024 Atropine 0.05%: +USD 1,993 Atropine 0.01%: +USD 1,931	France faster: −USD 7,585 (savings) UK faster: −USD 15,155 (savings) France slower: +USD 748 (higher) UK slower: ∼USD 0 (neutral)
Effectiveness and outcomes				
Effectiveness measure	Spherical equivalent distribution at adulthood (probabilistic); no QALY	QALYs: 0.013 gained/person screened 7 blindness cases prevented/100,000 screened	SER reduction (D): 0.05% = 3.42D; 0.01% = 1.98D AL reduction (mm): 0.05% = 1.80mm; 0.01% = 1.02mm	Spherical equivalent distribution at adulthood (probabilistic); no QALY
Utility measure	NR	QALYs (GBD disability weights): Myopia = 0.003; High myopia = 0.031; Pathological myopia = 0.184	NR	NR
Primary outcome metric	Lifetime cost (USD) Cost ratio ([AC]/[OC])	ICER: NZD per QALY gained	ICER: USD per diopter SER reduction, USD per mm AL reduction	Lifetime cost (USD) Cost ratio ([AC]/[OC])
Key results (Atropine)				
ICER [Cost ratio]	Australia : [cost ratio] ∼0.92 China : [cost ratio] ∼0.49	ICER = 2021 NZD 1,590/QALY (95% CI: NZD 1,390–1,791) 2024 USD = 1,188/QALY	Atropine 0.05%: ICER = USD 220/D SER (dominant strategy) Atropine 0.01%: ICER = USD 275/D SER Standardize 2024 Atropine 0.05%: ICER = USD 291/D SER Atropine 0.01%: ICER = USD 233/D SER	France faster: [cost ratio]= 0.77 (lifetime savings = USD 7,585) France slower: [cost ratio] = 1.03 UK faster: [cost ratio] = 0.69 (lifetime savings = USD 15,155) UK slower: [cost ratio] = 1.00 (neutral)
Adverse events costed	Not separately costed	Not separately costed (meta-analysis showed no serious AEs at 0.01%)	Yes — allergic conjunctivitis & photophobia included	Not separately costed
Convergent evidence synthesis				
Local CE threshold / Reference benchmark	Cost parity with [OC] (ratio = 1.0) No formal QALY threshold reported	NZD 58,000/QALY (WHO GDP/capita method)	Range USD 0–10,000/diopter SER or AL (study-defined novel threshold)	Cost parity with [OC] (ratio = 1.0) No formal QALY threshold reported
Position relative to threshold	Below [1.0] for in China; near neutral in Australia	2.7% of NZD 58,000 threshold; extremely cost-effective	2.2–2.75% of USD 10,000 upper threshold; extremely cost-effective	Below [1.0] for faster progressors in France and UK At or below parity for slower progressors in UK
Sensitivity analysis	Cost ranges; discount rates 0%, 3%, 5%; upper/lower limits; no PSA	Tornado diagram; PSA (Monte Carlo n=1,000); scenario analysis (5-yr to 95-yr life expectancy)	Deterministic & probabilistic sensitivity analyses; CEAC; WTP range USD 0–10,000/ diopter SER or AL	Cost ranges; 0%, 3% annual discounting; sex-disaggregated analysis; threshold price analysis for slower progressors
Main finding	Atropine = cheapest lifetime option in China; early investment intervention balanced; exceeded by lifetime savings	Screening + atropine highly cost-effective; 7 blindness cases prevented/100,000 screened; NNT to prevent 1 blindness = 14,286	Atropine 0.05% & outdoor activity are cost-effective; 0.05% = most cost-effective pharmacological strategy; 0.01% also cost-effective	Atropine reduces lifetime costs vs. OC for faster progressors (France & UK); cost-neutral to slightly higher for slower progressors; female individuals incur higher costs under OC, amplifying relative savings with atropine
Overall verdict	China - Cost-saving (0, 3%) Australia - Cost saving (3%);Cost-neutral (0%)	Highly cost-effective (ICER = 2.7% of local threshold)	Highly cost-effective (ICER = 2.2–2.75% of local range threshold)	Cost-saving (faster progressors, France & UK) Cost-neutral (slower progressors, UK)

1 Lifetime

2 5 year

For consistency, the intervention is described as 'atropine-based myopia control [AC]' and the comparator as 'conventional optical correction [OC]' across all studies. Original study-specific terminology: single-vision lenses (Agyekum et al., 2023b); usual care with corrective lenses (Hong et al., 2022); traditional myopia management—TMM (Fricke et al., 2023; Lee et al., 2025).

*Currency conversion: Costs from Fricke et al. (2023), Hong et al. (2022), and Agyekum et al. (2023b) were converted to 2024 USD using the CCEMG–EPPI Centre Cost Converter (https://eppi.ioe.ac.uk/costconversion/), applying country-specific GDP deflators and Purchasing Power Parities. Costs reported by Lee et al. (2025) were already expressed in 2024 USD and therefore required no further conversion.

Abbreviations: AL = axial length; AMM = active myopia management; CCA = cost-consequence analysis; CEA = cost-effectiveness analysis; CEAC = cost-effectiveness acceptability curve; CUA = cost-utility analysis; disc. = discounting; GBD = Global Burden of Disease; ICER = incremental cost-effectiveness ratio; NNT = number needed to treat; NR = not reported; PSA = probabilistic sensitivity analysis; QALY = quality-adjusted life year; SER = spherical equivalent refraction; TMM = traditional myopia management; WTP = willingness-to-pay.

Note on cross-study comparability: The three economic evaluation designs (CEA, CUA, CCA) serve distinct but complementary purposes. Direct quantitative pooling across designs is not appropriate and was not performed. A narrative convergent synthesis framework was applied, grouping studies by time horizon and evaluating each result relative to its local cost-effectiveness threshold or reference benchmark.

### 2.4 Risk of bias and quality assessment

The methodological quality of the included studies was assessed using both the Consolidated Health Economic Evaluation Reporting Standards (CHEERS) 2022 checklist (Husereau et al., 2022) and the model-based ECOBIAS checklist (Adarkwah et al., 2016). Each study was evaluated on key criteria outlined in the CHEERS checklist, including the clarity of the research question, detailed description of comparators, identification of relevant costs and outcomes, and the robustness of the economic model. Additionally, the ECOBIAS checklist was employed to assess the risk of bias in model-based economic evaluations, focusing on areas such as model assumptions, sensitivity analyses, and the handling of uncertainty. All quality assessments were conducted by two researchers and were validated by another two researchers independently. Responses to items in the critical appraisal checklist were tabulated and discussed in narrative form, with no summary score or overall statement (e.g., high or low quality). The completed CHEERS and ECOBIAS checklist are included in Supplementary Files S3 and S4, respectively.

## 3 Results and discussion

Our systematic search identified 374 potential studies. After duplicate removal, 280 unique records remained. Initial screening of titles and abstracts excluded 265 ineligible studies. We then performed full-text assessments of the remaining 15 articles using a predefined algorithm, which excluded, four studies lacking relevant outcome measures, one study available only as an abstract, five studies evaluating non-relevant interventions, two screening-only studies without therapeutic interventions.

A supplemental updated search on 15 May 2026 (PubMed and Google Scholar; 2025–2026) identified 77 additional records, from which one additional study was identified after screening, resulting in a total of 4 included studies from the two-time frame search (Hong et al., 2022; Fricke et al., 2023; Agyekum et al., 2023b; Lee et al., 2025). Tables 1 and 2 present the characteristics of the articles and summary of finding according to type of study.

**TABLE 2:** Cost ingredients captured by each included study.

Cost category	Fricke et al. (2023)	Hong et al. (2022)	Agyekum et al. (2023b)	Lee et al. (2025)
Perspective	Societal	Societal	Societal	Societal
Direct medical: examination / refraction	Yes	Yes	Yes	Yes
Optical correction / appliances	Yes	Yes (usual care)	Yes (lens changes)	Yes
Atropine / intervention cost	Yes	Yes	Yes	Yes
Monitoring / follow-up visits	Yes	Yes	Yes	Yes
Adverse-event management	No	No	Yes	No
Transportation / travel	Yes	No	No	Yes
Productivity / caregiver time	Yes	Yes	Yes	Yes
Downstream high-myopia complication costs	Yes	Yes	No (5-y horizon)	Yes
Atropine eye drop/month	China (USD1.50), Aus (USD21.52)	NZ (NZD 50)	NA	France (USD53.69), UK (USD84.23)
Reporting currency / price year	USD 2020	NZD 2021	USD 2022	USD 2024

Yes = cost category included in the study's costing; No = not included; NA = Not available. All studies adopted a nominally societal perspective, but the breadth of included cost ingredients varied; in particular, formal adverse-event management costs were captured only by Agyekum et al., and downstream high-myopia complication costs were captured only by the lifetime models (Fricke et al., 2023; Lee et al., 2025). This heterogeneity should be considered when interpreting the consistent cost-saving direction across studies.

Author reported: China is using pharmaceutical preparation, while other are compounded by pharmacy, Atropine in France were subsidize by government.

Note: Differences in atropine intervention categorisation across included studies should be considered when interpreting cross-study comparisons. Fricke et al. (2023) and Lee et al. (2025) modelled low-dose atropine as a grouped intervention, combining atropine concentrations ranging from 0.01% to 0.05% under a single low-dose atropine category within a cost-consequence analysis (CCA) framework. In contrast, Hong et al. (2022) evaluated atropine 0.01% alone as part of a photorefraction screening plus treatment strategy, whereby children screening positive for myopia received atropine in addition to standard optical correction. Agyekum et al. (2023b) analysed atropine 0.01% and atropine 0.05% as separate intervention arms, enabling direct comparison of concentration-specific cost-effectiveness relative to single-vision lenses. Consequently, variations in intervention definition, atropine concentration, and analytic framing may limit direct comparability of economic outcomes across studies.

### 3.1 Study characteristics

The included studies employed an analytical model approach using a hypothetical cohort of children aged 8 (Fricke et al., 2023; Lee et al., 2025), 10 (Agyekum et al., 2023b) and 11 (Hong et al., 2022) years, with populations model from China, Australia, Hong Kong, New Zealand, France, and the United Kingdom (UK).

Agyekum et al. (2023b) evaluated 13 myopia-control interventions using a cost-effectiveness framework over 5 years, including LDA (0.05% and 0.01%), defocus-incorporated multiple segment spectacles, contact lenses, orthokeratology, highly aspherical lenslets, and red-light therapy, with COC as the comparator, while Hong et al. (2022) constructed a cost-utility model to examine the incremental impact of adding photorefraction screening at age 11 and subsequent AT 0.01% treatment to OC. Meanwhile, Fricke et al. (2023) developed a cost-consequence model comparing OC with several active myopia management options, including LDA (as a group of 0.01%–0.05%), anti-myopia spectacles, multifocal soft contact lenses, and orthokeratology, using exemplar cohorts from Australia and China. The recent publication by Lee et al. (2025) extended the Fricke et al. (2023) cost-consequence methodology to European settings (France and UK), across separate progression risk profiles (faster and slower progressors).

All studies were funded by research grants and adopted a societal perspective (Hong et al., 2022; Fricke et al., 2023; Agyekum et al., 2023b; Lee et al., 2025). A discount rate of 3% was applied across studies, with sensitivity analyses using 0% (Fricke et al., 2023), 5% (Fricke et al., 2023) and 6% (Hong et al., 2022); only Hong et al. (2022) explicitly discounted both costs and utilities. The modeled interventions spanned 5 years, with time horizons ranging from 5 years (Agyekum et al., 2023b) to a lifetime (Hong et al., 2022; Fricke et al., 2023; Lee et al., 2025).

### 3.2 Economic analytical model types

Two studies employed Markov cohort state-transition models, whereas two used a probabilistic cohort decision-analytic model without explicit health states. Agyekum et al. (2023b) implemented a three-state Markov model (low, moderate, and high myopia) over a 5-year horizon, with irreversible progression governed by intervention-specific changes in spherical equivalent and AL progression rates; outcomes were reported as cost per diopter and per millimeter AL reduction.

Hong et al. (2022) developed a hybrid decision-tree Markov model incorporating photorefraction screening at age 11 followed by a five-state Markov process (normal vision, myopia, high myopia, pathological myopia, and blindness/visual impairment) extending to a lifetime horizon, enabling cost-utility analysis measured in quality-adjusted life years (QALYs).

Conversely, Fricke et al. (2023) and Lee et al. (2025) utilized probabilistic cohort models based on continuous refractive progression trajectories from childhood to early adulthood. These models generated probability distributions of adult refractive outcomes that informed lifetime cost estimates for refractive correction and vision-threatening complications, relying on cost ratios and total lifetime financial savings rather than estimating QALYs or formal incremental cost-effectiveness ratios (ICERs).

Although heterogeneous in structure, time horizon, and outcome metrics, these models collectively capture both the short-term clinical intervention effects and the massive long-term economic consequences of myopia progression.

### 3.3 Risk of bias and quality assessment

#### 3.3.1 ECOBIAS checklist for model-based economic evaluations

The table in Supplementary Material offers a comprehensive analysis of potential biases in model-based economic evaluations using the ECOBIAS checklist. All four studies included in the analysis adopted a societal perspective, ensuring that all relevant costs were considered, including medical costs and productivity loss (Hong et al., 2022; Fricke et al., 2023; Agyekum et al., 2023b; Lee et al., 2025). However, there were some limitations noted, such as unclear descriptions regarding intermittent data collection and the absence of detailed treatment protocols, which could pose risks of bias.

Additionally, the valuation of prices was clearly presented, and the studies adhered to the correct discounting rates of 3%, which mitigates inappropriate discounting bias. All four studies chose broadly appropriate decision-analytic structures for the research question (two Markov cohort models and two cohort decision-analytic cost model), although Fricke et al. (2023) and Lee et al. (2025) did not implement an explicit health-state transition framework and reported costs only, which limits comparability with standard cost-utility models.

In terms of treatment effects and data incorporation, all studies utilized relevant data from high-quality sources and employed appropriate meta-analytic technique. However, the absence of utility values in three studies (Fricke et al., 2023; Agyekum et al., 2023b; Lee et al., 2025) and limited transparency in reporting treatment protocols could limit the robustness of the evaluations.

Overall, while the studies demonstrate substantial rigor in terms of model structure and cost inclusion, areas such as data collection methods, reporting transparency, and utility incorporation require further attention to reduce potential biases in the economic evaluations.

#### 3.3.2 CHEERS reporting guidelines assessment

While all four included studies utilized the Consolidated Health Economic Evaluation Reporting Standards (CHEERS) guidelines to structure their reporting, there was notable heterogeneity in their comprehensiveness and chosen outcome metrics. Hong et al. (2022) adhered to nearly all CHEERS criteria apart from providing a pre-specified health economic analysis plan (Criteria 4); ensuring that essential elements such as the study’s societal perspective, comparator choices, and comprehensive cost considerations were clearly described. Unlike standard practice in health economic evaluations, Agyekum et al. (2023b) did not report QALYs; instead, their analysis focused strictly on SER and AL outcomes. Despite their clinical relevance, these outcomes limit comparison with other economic studies.

Similarly, Fricke et al. (2023) and Lee et al. (2025) did not estimate QALYs or ICERs with Fricke et al. (2023)’s reporting exhibited minor limitations in line with strict CHEERS standards, such as a less explicit title regarding the specific economic evaluation type and mapping the lifelong consequences of the disease primarily through visual probability profiles rather than explicit health states. The overall methodological quality of the articles was deemed adequate, and transparency was maintained across the literature, as all four studies explicitly declared receiving financial funding.

### 3.4 Sources of the model input

#### 3.4.1 Type of cost

All four studies captured direct medical costs, although the scope, time horizon, and perspective of their costing differed. Fricke et al. (2023) included direct medical costs alongside productivity losses and long-term complication costs, encompassing examinations, refraction, optical correction, active myopia management, and management of complications, with costs reported in 2020 US Dollars (USD). Similarly, Lee et al. (2025) evaluated lifetime societal costs in France and the UK, incorporating direct medical expenses, optical appliances, and indirect costs such as transportation and productivity loss for both patients and caregivers, with costs converted to 2024 USD.

The remaining two studies utilized different frameworks. Agyekum et al. (2023b) reported both direct and indirect costs over a 5-year horizon, including consultations, refraction, lens changes, adverse event management, and caregiver time or productivity loss, with values expressed in 2022 USD. Finally, Hong et al. (2022) focused on direct medical costs related to a hypothetical screening program, atropine-related clinic visits, monitoring tests, and low vision or blindness care, alongside productivity losses, with costs presented in 2021 New Zealand Dollars (NZD).

#### 3.4.2 Cost of atropine eye drops (AEDs)

The cost of atropine eye drops (AEDs) varied markedly across countries and concentrations. This variation appeared to be influenced by regional drug pricing, healthcare subsidies, and differences in formulation availability [Refer Table 2].

The lowest reported cost was in mainland China, where AEDs was priced at USD 1.50 per month, or USD 18 annually. In Australia, compounded AEDs at concentrations of 0.01%–0.05% cost USD 21.52 per month, equivalent to USD 258.24 per year. In Europe, a 1-month supply was estimated at USD 53.69 in France, where treatment was subsidized, and USD 84.23 in the United Kingdom, where it was paid out of pocket.

In New Zealand, the cost of compounded 0.01% AEDs was reported at approximately NZD50 per month, reflecting local compounding pricing practices (Hong et al., 2022). While Hong Kong study did not report the unit or monthly cost of AEDs, and therefore no specific price could be extracted from that analysis (Agyekum et al., 2023b). Notably, Fricke et al. (2023); Lee et al. (2025) grouped atropine concentrations of 0.01%–0.05% into a single intervention category for cost estimation whereas Agyekum et al. (2023b) reported separate cost estimates for 0.01% and 0.05% atropine.

Treatment duration for LDA differed across models, ranging from a fixed 5-year course in Agyekum et al. (2023b) to 10 years in Fricke et al. (2023), intermittent 2-year treatment and one year-off cycles through age 17 in Lee et al. (2025), and 2–3 years of 0.01% atropine after screening in Hong et al. (2022) Across the included economic evaluations, atropine was never costed as the eye drop alone; rather, it was embedded within a package of care that typically included prescribing, follow-up, refraction or monitoring visits, and in some studies, transport and productivity losses. Fricke et al. (2023) and Lee et al. (2025) adopted lifetime societal models that linked atropine use to broader ophthalmic care pathways and future complication costs, whereas Agyekum et al. (2023b) estimated 5-year costs for atropine 0.01% and 0.05% separately in Hong Kong, and Hong et al. (2022) evaluated atropine 0.01% within a screening programmed in New Zealand.

#### 3.4.3 Effectiveness outcomes

The effectiveness of atropine was evaluated across all included studies using SER as the primary outcome measure. Agyekum et al. (2023b) additionally incorporated axial length (AL) as a secondary parameter in their analysis. Among the reviewed studies, only one reported QALYs as an outcome metric (Hong et al., 2022). All studies derived their effectiveness inputs from systematic reviews and meta-analyses (Hong et al., 2022; Fricke et al., 2023; Agyekum et al., 2023b), with the Atropine for the Treatment of Myopia-2 (ATOM2) trial serving as the pivotal reference for clinical efficacy data (Chia et al., 2016). The single study reporting QALYs calculated utilities by inverting disability weights from the Global Burden of Disease (GBD) Study, assigning values of 0.003 for myopia, 0.031 for high myopia, and 0.184 for pathological myopia (Hong et al., 2022).

Effectiveness data were primarily based on SER and AL outcomes, with limited reporting of QALYs. The ATOM2 (Chia et al., 2016) trial served as the cornerstone for efficacy estimates, while utility values were derived from GBD disability weights. These metrics provide a foundation for assessing both clinical and economic impact of atropine treatment.

#### 3.4.4 Adverse event considerations

Notably, Agyekum et al. (2023b) was the sole study to incorporate costs associated with treating adverse effects of atropine, specifically allergic conjunctivitis and photophobia. Other analyses omitted economic inputs related to side-effect management (Hong et al., 2022; Fricke et al., 2023).

#### 3.4.5 Cost-effectiveness outcomes

Despite heterogeneity in economic model design encompassing cost-utility analysis (CUA), cost-effectiveness analysis (CEA), and cost-consequence analysis (CCA). All four included studies demonstrated a consistent directional finding: AT is cost-effective, or cost-saving compared with OC. Direct quantitative pooling across studies was not performed due to fundamental differences in outcome metrics (QALYs, diopters of SER reduction, lifetime costs, and cost ratios), time horizons (5 years to lifetime), and healthcare system contexts. Findings are therefore synthesised narratively in accordance with established guidance for systematic reviews of heterogeneous economic evaluations. To facilitate cross-study comparison, Table 1 presents each result in relation to its local cost-effectiveness threshold or reference cost benchmark, enabling a standardised appraisal of the magnitude of economic benefit across heterogeneous designs.

Economic advantage was consistently greatest for children at risk of faster myopia progression, where avoidance of high-myopia complications substantially reduces lifetime healthcare and productivity costs. Direct quantitative pooling across studies was not performed due to differences in outcome metrics, time horizons, and healthcare system contexts; findings are therefore synthesized narratively in accordance with established guidance for systematic reviews of heterogeneous economic evaluations.

### 3.5 Assessing uncertainty in cost-effectiveness result

The four included studies all explored uncertainty, although with varying depth and transparency. Agyekum et al. (2023b) used both deterministic and probabilistic sensitivity analyses and tested key structural assumptions such as starting age, discount rate, and time horizon across clinically plausible ranges, with gamma distributions for costs and normal distributions for treatment effects. Fricke et al. (2023) and the later European adaptation by Lee et al. (2025) approached uncertainty primarily through upper- and lower-limit cost estimates, alternative discount rates, and threshold analyses examining the monthly atropine price at which active myopia management would become cost-neutral relative to traditional care. Hong et al. (2022) provided the most comprehensive uncertainty analysis, combining one-way sensitivity analysis, tornado diagrams, scenario analyses, and probabilistic sensitivity analysis, and showed that the ICER was most sensitive to pathological myopia costs, discount rate, and atropine efficacy. Despite these methodological differences, all four studies showed that low-dose atropine remained economically favorable across plausible assumptions, supporting the robustness of the overall conclusion.

### 3.6 Cost-effectiveness thresholds

Cost-effectiveness thresholds and ICERs used in the four studies are summarised in Table 1. Hong et al. (2022) judged screening plus atropine against a conventional GDP-per-capita QALY threshold, whereas Agyekum et al. (2023b) applied willingness-to-pay ranges per unit SER/AL change, and Fricke et al. (2023) and Lee et al. (2025) focused on lifetime cost ratios and threshold drug prices rather than formal ICER thresholds. These differences in threshold frameworks should be considered when comparing results across settings or translating them to other country, where QALY-based thresholds of 1–3 × GDP *per capita* would be more appropriate (World Health Organization, 2001).

Given the heterogeneity in model structure, outcome measures, time horizons, and underlying assumptions across the included studies, the overall interpretation was based primarily on the direction of effect, that is, whether LDA was consistently associated with favorable economic outcomes relative to comparator care rather than on direct comparison of threshold values or ICER magnitudes across studies.

### 3.7 Discussion and recommendations for future research

This review found that LDA is generally a cost-effective strategy for pediatric myopia control, although the magnitude of benefit varies across settings because each model is shaped by different assumptions on treatment duration, care pathways, and local prices. Across the four included economic evaluations, atropine was assessed in Hong Kong, Australia/China, New Zealand, and the United Kingdom/France, with all studies using a societal perspective and standard single-vision correction as the reference case. Together, these studies suggest that investment in childhood myopia control may be offset later in life through lower refractive progression, fewer complex optical needs, and a reduced risk of high-myopia complications.

The alignment of the hypothetical cohorts (ages 8–11 years) with the age range of RCT participants (6–12 years) (Zaabaar et al., 2024) enhances the external validity of this review’s findings, as the modeled demographics closely mirror the clinical trial populations in which atropine’s efficacy was empirically established (Chia et al., 2016; Yam et al., 2019).

A major source of heterogeneity was the assumed atropine regimen, with modeled treatment durations ranging from approximately 2–10 years across four studies. This variation is clinically reassuring rather than problematic, because despite differing assumptions about how long children remain on therapy and how frequently they are reviewed, all models still converged on low-dose atropine as a cost-effective option for myopia control in their respective settings.

We found that AEDs themselves represent only one component of total cost with follow-up visits, refraction, monitoring, and downstream management of high-myopia complications contributed substantially to total expenditure, particularly in lifetime models. Since atropine is modeled as a finite treatment rather than lifelong therapy, the additional cost of ophthalmologist or optometrist review is concentrated during childhood, whereas long-term savings are driven mainly by lower adult myopia severity and fewer pathological outcomes. This helps explain why lifetime models generally show greater economic benefit than shorter-horizon analyses.

Cross-country differences in the pricing and delivery of AEDs further limit direct comparisons between studies. Fricke et al. (2023) reported substantially lower atropine costs in China compared with Australia, while Agyekum et al. (2023b) found that 0.05% atropine in Hong Kong demonstrated a more favorable cost-effectiveness profile than 0.01%. Notably, these findings were generated during a period when LDA was primarily available as compounded formulations, which may have influenced both pricing and accessibility. As licensed pharmaceutical formulations enter practice (Medsafe, 2023; Optometry Australia, 2024), older estimates based on compounding may no longer reflect real-world acquisition costs, dispensing pathways, or reimbursement arrangements.

Another important consideration is the role of healthcare providers in prescribing and monitoring atropine therapy. In many healthcare systems, atropine remains closely linked to ophthalmologist-led care (Yap and Mishu, 2024), whereas optical correction and myopia progression monitoring may be delivered in optometric settings. In addition, government subsidies, such as those implemented in France, are likely to increase uptake compared with settings where such treatments are not subsidized.

This distinction matters because systems requiring both ophthalmology and optometry visits may incur higher costs and greater inconvenience, as highlighted in regional policy analyses such as Yap and Mishu (2024). Therefore, although the current evidence supports atropine as an economically promising intervention, transferability to Malaysia and other ASEAN settings requires caution and should be interpreted against local service-delivery models and cost-effectiveness thresholds relevant to middle-income health systems.

Future studies should validate current model assumptions using long-term real-world data, particularly on treatment duration, rebound after cessation, and the lifetime impact of preventing high myopia. Economic models should also be updated to reflect the growing availability of licensed pharmaceutical low-dose atropine products, since approved agents such as Eikance in New Zealand (Medsafe, 2023) and Australia (Optometry Australia, 2024), and Ryjunea in the European (European Medicines Agency, 2025) countries may have different prices and reimbursement pathways from compounded formulations.

In addition, future CEAs should compare ophthalmologist-led, optometrist-led, and shared-care pathways, and include the full cost of follow-up, adverse-event management, and caregiver time.

## 4 Conclusion

This systematic review provides the first focused synthesis of the economic evaluations of atropine eye drops for childhood myopia management. Despite the limited number of studies and methodological heterogeneity, the evidence consistently supports low-dose atropine (0.01%–0.05%) as a cost-effective or cost-saving intervention compared to conventional management strategies, such as single-vision lenses or no treatment. Lifetime modelling further highlight the economic advantage of atropine by preventing costly high myopia complications and reducing long-term productivity losses. While atropine represents a promising, economically viable intervention for reducing the global burden of childhood myopia, its broader consideration in public health strategies warrants thorough evaluation of local context and long-term real-world outcomes.

## Data Availability

The original contributions presented in the study are included in the article/Supplementary Material, further inquiries can be directed to the corresponding author.
