# Supplementary material for: Systematic review of atropine cost-effectiveness in childhood myopia management
Source: Front Pharmacol. 2026 Oct 16;17:1836579. doi: 10.3389/fphar.2026.1836579 (PMC13720285; doi:10.3389/fphar.2026.1836579)
Supplement: Supplementary file 1 [file Table1.docx]

**Search strategy**

**Database PubMed**

| **Search number** | **Query** | **Sort By** | **Filters** | **Search Details** | **Results** | **Time** |
| --- | --- | --- | --- | --- | --- | --- |
| **12** | #9 AND #10 |  | from 2022/1/1 - 2025/4/1 | (("cost*"[All Fields] OR ("cost benefit analysis"[MeSH Terms] OR ("cost benefit"[All Fields] AND "analysis"[All Fields]) OR "cost benefit analysis"[All Fields] OR ("cost"[All Fields] AND "effectiveness"[All Fields]) OR "cost effectiveness"[All Fields]) OR ("cost benefit analysis"[MeSH Terms] OR ("cost benefit"[All Fields] AND "analysis"[All Fields]) OR "cost benefit analysis"[All Fields] OR ("economic"[All Fields] AND "evaluation"[All Fields]) OR "economic evaluation"[All Fields])) AND ("myopia"[MeSH Terms] OR "myopia"[All Fields] OR "myopias"[All Fields] OR "nearsight*"[All Fields] OR "shortsight*"[All Fields]) AND ("Atropine"[MeSH Terms] OR "ophthalmic solutions"[MeSH Terms] OR ("ophthalmic solutions"[All Fields] OR "eye drop"[Text Word]) OR ("medical intervention"[All Fields] OR ("medic"[All Fields] OR "medical"[All Fields] OR "medicalization"[MeSH Terms] OR "medicalization"[All Fields] OR "medicalizations"[All Fields] OR "medicalize"[All Fields] OR "medicalized"[All Fields] OR "medicalizes"[All Fields] OR "medicalizing"[All Fields] OR "medically"[All Fields] OR "medicals"[All Fields] OR "medicated"[All Fields] OR "medication s"[All Fields] OR "medics"[All Fields] OR "pharmaceutical preparations"[MeSH Terms] OR ("pharmaceutical"[All Fields] AND "preparations"[All Fields]) OR "pharmaceutical preparations"[All Fields] OR "medication"[All Fields] OR "medications"[All Fields]) OR ("intervention s"[All Fields] OR "interventions"[All Fields] OR "interventive"[All Fields] OR "methods"[MeSH Terms] OR "methods"[All Fields] OR "intervention"[All Fields] OR "interventional"[All Fields])))) AND (2022/1/1:2025/4/1[pdat]) | 102 | 03:12:03 |
| **11** | #9 AND #10 |  |  | ("cost*"[All Fields] OR ("cost benefit analysis"[MeSH Terms] OR ("cost benefit"[All Fields] AND "analysis"[All Fields]) OR "cost benefit analysis"[All Fields] OR ("cost"[All Fields] AND "effectiveness"[All Fields]) OR "cost effectiveness"[All Fields]) OR ("cost benefit analysis"[MeSH Terms] OR ("cost benefit"[All Fields] AND "analysis"[All Fields]) OR "cost benefit analysis"[All Fields] OR ("economic"[All Fields] AND "evaluation"[All Fields]) OR "economic evaluation"[All Fields])) AND ("myopia"[MeSH Terms] OR "myopia"[All Fields] OR "myopias"[All Fields] OR "nearsight*"[All Fields] OR "shortsight*"[All Fields]) AND ("Atropine"[MeSH Terms] OR "ophthalmic solutions"[MeSH Terms] OR ("ophthalmic solutions"[All Fields] OR "eye drop"[Text Word]) OR ("medical intervention"[All Fields] OR ("medic"[All Fields] OR "medical"[All Fields] OR "medicalization"[MeSH Terms] OR "medicalization"[All Fields] OR "medicalizations"[All Fields] OR "medicalize"[All Fields] OR "medicalized"[All Fields] OR "medicalizes"[All Fields] OR "medicalizing"[All Fields] OR "medically"[All Fields] OR "medicals"[All Fields] OR "medicated"[All Fields] OR "medication s"[All Fields] OR "medics"[All Fields] OR "pharmaceutical preparations"[MeSH Terms] OR ("pharmaceutical"[All Fields] AND "preparations"[All Fields]) OR "pharmaceutical preparations"[All Fields] OR "medication"[All Fields] OR "medications"[All Fields]) OR ("intervention s"[All Fields] OR "interventions"[All Fields] OR "interventive"[All Fields] OR "methods"[MeSH Terms] OR "methods"[All Fields] OR "intervention"[All Fields] OR "interventional"[All Fields]))) | 350 | 03:11:09 |
| **10** | ("Atropine"[Mesh]) OR ("ophthalmic solutions"[MeSH Terms]) OR ("ophthalmic solutions"[All Fields] OR eye drop[Text Word]) OR ("medical intervention" OR medical OR intervention) |  |  | "Atropine"[MeSH Terms] OR "ophthalmic solutions"[MeSH Terms] OR ("ophthalmic solutions"[All Fields] OR "eye drop"[Text Word]) OR ("medical intervention"[All Fields] OR ("medic"[All Fields] OR "medical"[All Fields] OR "medicalization"[MeSH Terms] OR "medicalization"[All Fields] OR "medicalizations"[All Fields] OR "medicalize"[All Fields] OR "medicalized"[All Fields] OR "medicalizes"[All Fields] OR "medicalizing"[All Fields] OR "medically"[All Fields] OR "medicals"[All Fields] OR "medicated"[All Fields] OR "medication s"[All Fields] OR "medics"[All Fields] OR "pharmaceutical preparations"[MeSH Terms] OR ("pharmaceutical"[All Fields] AND "preparations"[All Fields]) OR "pharmaceutical preparations"[All Fields] OR "medication"[All Fields] OR "medications"[All Fields]) OR ("intervention s"[All Fields] OR "interventions"[All Fields] OR "interventive"[All Fields] OR "methods"[MeSH Terms] OR "methods"[All Fields] OR "intervention"[All Fields] OR "interventional"[All Fields])) | 16,451,728 | 03:09:57 |
| **9** | #4 AND #8 |  |  | ("cost*"[All Fields] OR ("cost benefit analysis"[MeSH Terms] OR ("cost benefit"[All Fields] AND "analysis"[All Fields]) OR "cost benefit analysis"[All Fields] OR ("cost"[All Fields] AND "effectiveness"[All Fields]) OR "cost effectiveness"[All Fields]) OR ("cost benefit analysis"[MeSH Terms] OR ("cost benefit"[All Fields] AND "analysis"[All Fields]) OR "cost benefit analysis"[All Fields] OR ("economic"[All Fields] AND "evaluation"[All Fields]) OR "economic evaluation"[All Fields])) AND ("myopia"[MeSH Terms] OR "myopia"[All Fields] OR "myopias"[All Fields] OR "nearsight*"[All Fields] OR "shortsight*"[All Fields]) | 455 | 03:08:58 |
| **8** | #5 OR #6 OR #7 |  |  | "myopia"[MeSH Terms] OR "myopia"[All Fields] OR "myopias"[All Fields] OR "nearsight*"[All Fields] OR "shortsight*"[All Fields] | 33,324 | 03:08:33 |
| **7** | "shortsight*"[All Fields] |  |  | "shortsight*"[All Fields] | 498 | 03:08:08 |
| **6** | "nearsight*"[All Fields] |  |  | "nearsight*"[All Fields] | 278 | 03:08:00 |
| **5** | "myopia"[MeSH Terms] OR "myopia"[All Fields] OR "myopias"[All Fields] |  |  | "myopia"[MeSH Terms] OR "myopia"[All Fields] OR "myopias"[All Fields] | 32,829 | 03:07:42 |
| **4** | #1 OR #2 OR #3 |  |  | "cost*"[All Fields] OR ("cost benefit analysis"[MeSH Terms] OR ("cost benefit"[All Fields] AND "analysis"[All Fields]) OR "cost benefit analysis"[All Fields] OR ("cost"[All Fields] AND "effectiveness"[All Fields]) OR "cost effectiveness"[All Fields]) OR ("cost benefit analysis"[MeSH Terms] OR ("cost benefit"[All Fields] AND "analysis"[All Fields]) OR "cost benefit analysis"[All Fields] OR ("economic"[All Fields] AND "evaluation"[All Fields]) OR "economic evaluation"[All Fields]) | 1,176,380 | 03:07:20 |
| **3** | "cost benefit analysis"[MeSH Terms] OR ("cost benefit"[All Fields] AND "analysis"[All Fields]) OR "cost benefit analysis"[All Fields] OR ("economic"[All Fields] AND "evaluation"[All Fields]) OR "economic evaluation"[All Fields] |  |  | "cost benefit analysis"[MeSH Terms] OR ("cost benefit"[All Fields] AND "analysis"[All Fields]) OR "cost benefit analysis"[All Fields] OR ("economic"[All Fields] AND "evaluation"[All Fields]) OR "economic evaluation"[All Fields] | 139,459 | 03:06:58 |
| **2** | "cost benefit analysis"[MeSH Terms] OR ("cost benefit"[All Fields] AND "analysis"[All Fields]) OR "cost benefit analysis"[All Fields] OR ("cost"[All Fields] AND "effectiveness"[All Fields]) OR "cost effectiveness"[All Fields] |  |  | "cost benefit analysis"[MeSH Terms] OR ("cost benefit"[All Fields] AND "analysis"[All Fields]) OR "cost benefit analysis"[All Fields] OR ("cost"[All Fields] AND "effectiveness"[All Fields]) OR "cost effectiveness"[All Fields] | 173,873 | 03:06:38 |
| **1** | cost* |  |  | "cost*"[All Fields] | 1,151,420 | 03:05:05 |

**Database: Embase <1974 to 2025 April 04>**
**Search Strategy:**
**1**  "cost benefit analysis"/ or "cost effectiveness analysis"/ or "cost utility analysis"/ (293480)
**2**  ("cost benefit analysis" or "cost effectiveness analysis" or "cost utility analysis").af. (296461)
**3**  ("cost benefit analysis" or "cost effectiveness analysis" or "cost utility analysis" or (economic evaluation or "cost benefit analysis" or "cost effectiveness analysis" or "cost")).af. (1212950)
**4**  (economic evaluation or "cost benefit analysis" or "cost effectiveness analysis" or "cost").af. (1212950)
**5**  ("economic evaluation" or "cost benefit analysis" or "cost effectiveness analysis" or "cost").af. (1212950)
**6**  ("cost benefit analysis" or "cost effectiveness analysis" or "cost utility analysis").af. (296461)
**7**  ("economic evaluation" or "cost").af. (1212950)

**Concept finalized**
**8**  ("cost benefit analysis" or "cost effectiveness analysis" or "cost utility analysis" or "economic evaluation" or "cost").af. (1212950)
**9**  (myopia* or high myopia).af. (43766)
**10**  (("cost benefit analysis" or "cost effectiveness analysis" or "cost utility analysis" or "economic evalution" or "cost") and (myopia* or high myopia)).af. (544)

Filter 2022-2025 :144

**Database: ProQuest**

Set#: S1

Searched for: cost

Databases: Research Library, Science Database

Results: 11212897

Set#: S2

Searched for: cost-effectiveness

Databases: Research Library, Science Database

Results: 171524

Set#: S3

Searched for: economic evaluation/analysis

Databases: Research Library, Science Database

Results: 965

Set#: S4

Searched for: myopia

Databases: Research Library, Science Database

Results: 31072

Set#: S5

Searched for: nearsightedness

Databases: Research Library, Science Database

Results: 2094

Set#: S6

Searched for: shortsightedness

Databases: Research Library, Science Database

Results: 3957

Set#: S7

Searched for: [S1] OR [S2] OR [S3]

Databases: Research Library, Science Database

These databases are searched for part of your query.

Results: 11213268

Set#: S8

Searched for: [S4] OR [S5] OR [S6]

Databases: Research Library, Science Database

These databases are searched for part of your query.

Results: 35941

Set#: S9

Searched for: [S7] AND [S8]

Databases: Research Library, Science Database

These databases are searched for part of your query.

Results: 11212

Set#: S10

Searched for: (medical intervention) OR atropine OR (eye drops)

Databases: Research Library, Science Database

Results: 1185864

Set#: S11

Searched for: [S9] AND [S10]

Databases: Research Library, Science Database

These databases are searched for part of your query.

Results: 3034

Set#: S12

Searched for: [S9] AND [S10] AND subt.exact("studies" OR "decision making" OR "human" OR "ophthalmology" OR "myopia")

Databases: Research Library, Science Database

These databases are searched for part of your query.

Results: 678

Set#: S13

Searched for: [S12] AND la.exact("ENG")

Databases: Research Library, Science Database

These databases are searched for part of your query.

Results: 676

Set#: S14

Searched for: "Magazines" OR "Reports" OR "Wire Feeds" OR "Blogs, Podcasts, & Websites" OR "Working Papers"

Databases: Research Library, Science Database

Results: 7096449

Set#: S15

Searched for: [S13] NOT [S14]

Databases: Research Library, Science Database

These databases are searched for part of your query.

Results: 346

Set#: S16

Searched for: ([S13] NOT [S14]) AND pd(20220101-20250406)

Databases: Research Library, Science Database

These databases are searched for part of your query.

Results: 56

| **Database: Web of Science Core Collection** | |
| --- | --- |
| 1: ALL=(cost) | Date Run: Sun Apr 06 2025 16:27:52 GMT+0800 (Malaysia Time) Results: 2689514 |
|  |  |
| 2: ALL=(cost-effective*) | Date Run: Sun Apr 06 2025 16:28:40 GMT+0800 (Malaysia Time) Results: 425181 |
|  |  |
| 3: ALL=(economic evaluation) | Date Run: Sun Apr 06 2025 16:29:07 GMT+0800 (Malaysia Time) Results: 143679 |
|  |  |
| 4: #1 OR #2 OR #3 | Date Run: Sun Apr 06 2025 16:29:26 GMT+0800 (Malaysia Time) Results: 2785010 |
|  |  |
| 5: ALL=(myopia) | Date Run: Sun Apr 06 2025 16:29:59 GMT+0800 (Malaysia Time) Results: 30640 |
|  |  |
| 6: ALL=(shortsight*) | Date Run: Sun Apr 06 2025 16:30:22 GMT+0800 (Malaysia Time) Results: 536 |
|  |  |
| 7: ALL=(nearsight*) | Date Run: Sun Apr 06 2025 16:31:08 GMT+0800 (Malaysia Time) Results: 312 |
|  |  |
| 8: #5 OR #6 OR #7 | Date Run: Sun Apr 06 2025 16:31:22 GMT+0800 (Malaysia Time) Results: 31337 |
|  |  |
| 9: #4 AND #8 | Date Run: Sun Apr 06 2025 16:31:34 GMT+0800 (Malaysia Time) Results: 655 |
|  |  |
| 10: ALL=(medical intervention) | Date Run: Sun Apr 06 2025 16:32:08 GMT+0800 (Malaysia Time) Results: 1051178 |
|  |  |
| 11: ALL=(atropine) | Date Run: Sun Apr 06 2025 16:32:28 GMT+0800 (Malaysia Time) Results: 32769 |
|  |  |
| 12: ALL=(eye drop) | Date Run: Sun Apr 06 2025 16:32:51 GMT+0800 (Malaysia Time) Results: 18704 |
|  |  |
| 13: #10 OR #11 OR #12 | Date Run: Sun Apr 06 2025 16:33:06 GMT+0800 (Malaysia Time) Results: 1100991 |
|  |  |
| 14: #9 AND #13 | Date Run: Sun Apr 06 2025 16:33:25 GMT+0800 (Malaysia Time) Results: 58 |
|  |  |

**Scopus 26/3/2025**

A. **Cost-related terms**

( TITLE-ABS-KEY ( "economic evaluation" ) OR TITLE-ABS-KEY ( "cost effectiveness" ) OR TITLE-ABS-KEY ( "cost benefit" ) )

639,797 results

( TITLE-ABS-KEY ( cost* ) AND TITLE-ABS-KEY ( analysis ) AND TITLE-ABS-KEY ( effectiveness ) AND TITLE-ABS-KEY ( benefit ) )

103,121 results

B. **Myopia-Related Terms**

( TITLE-ABS-KEY ( myopia ) OR TITLE-ABS-KEY ( myopias ) OR TITLE-ABS-KEY ( shortsighted* ) OR TITLE-ABS-KEY ( nearsighted* ) )

48,722 results

**A and B**

( ( TITLE-ABS-KEY ( myopia ) OR TITLE-ABS-KEY ( myopias ) OR TITLE-ABS-KEY ( shortsighted* ) OR TITLE-ABS-KEY ( nearsighted* ) ) ) AND ( ( TITLE-ABS-KEY ( cost* ) AND TITLE-ABS-KEY ( analysis ) AND TITLE-ABS-KEY ( effectiveness ) AND TITLE-ABS-KEY ( benefit ) ) ) AND ( ( TITLE-ABS-KEY ( "economic evaluation" ) OR TITLE-ABS-KEY ( "cost effectiveness" ) OR TITLE-ABS-KEY ( "cost benefit" ) ) )

Result : 53

Filter 2022-2025 :14

Updated supplemental search with PubMed and Google Scholar 15 May 2026 [From 2025 – 2026] = 77

| **Search number** | **Query** | **Sort By** | **Filters** | **Search Details** | **Results** | **Time** | **Date** |
| --- | --- | --- | --- | --- | --- | --- | --- |
| **9** | ((("cost*"[All Fields] AND (y_1[Filter])) OR ("cost benefit analysis"[MeSH Terms] OR ("cost benefit"[All Fields] AND "analysis"[All Fields]) OR "cost benefit analysis"[All Fields] OR ("cost"[All Fields] AND "effectiveness"[All Fields]) OR "cost effectiveness"[All Fields] AND (y_1[Filter]))) OR ("cost benefit analysis"[MeSH Terms] OR ("cost benefit"[All Fields] AND "analysis"[All Fields]) OR "cost benefit analysis"[All Fields] OR ("economic"[All Fields] AND "evaluation"[All Fields]) OR "economic evaluation"[All Fields] AND (y_1[Filter])) AND (y_1[Filter])) AND ((("myopia"[MeSH Terms] OR "myopia"[All Fields] OR "myopias"[All Fields] AND (y_1[Filter])) OR ("nearsight*"[All Fields] AND (y_1[Filter]))) OR ("shortsight*"[All Fields] AND (y_1[Filter])) AND (y_1[Filter])) |  | in the last 1 year | ((("cost*"[All Fields] AND "2025/05/15 00:00":"3000/01/01 05:00"[Date - Publication]) OR (("cost benefit analysis"[MeSH Terms] OR ("cost benefit"[All Fields] AND "analysis"[All Fields]) OR "cost benefit analysis"[All Fields] OR ("cost"[All Fields] AND "effectiveness"[All Fields]) OR "cost effectiveness"[All Fields]) AND "2025/05/15 00:00":"3000/01/01 05:00"[Date - Publication]) OR (("cost benefit analysis"[MeSH Terms] OR ("cost benefit"[All Fields] AND "analysis"[All Fields]) OR "cost benefit analysis"[All Fields] OR ("economic"[All Fields] AND "evaluation"[All Fields]) OR "economic evaluation"[All Fields]) AND "2025/05/15 00:00":"3000/01/01 05:00"[Date - Publication])) AND "2025/05/15 00:00":"3000/01/01 05:00"[Date - Publication] AND (((("myopia"[MeSH Terms] OR "myopia"[All Fields] OR "myopias"[All Fields]) AND "2025/05/15 00:00":"3000/01/01 05:00"[Date - Publication]) OR ("nearsight*"[All Fields] AND "2025/05/15 00:00":"3000/01/01 05:00"[Date - Publication]) OR ("shortsight*"[All Fields] AND "2025/05/15 00:00":"3000/01/01 05:00"[Date - Publication])) AND "2025/05/15 00:00":"3000/01/01 05:00"[Date - Publication])) AND (y_1[Filter]) | 69 | 01:57:01 | 2026/05/15 |
| **8** | (("myopia"[MeSH Terms] OR "myopia"[All Fields] OR "myopias"[All Fields] AND (y_1[Filter])) OR ("nearsight*"[All Fields] AND (y_1[Filter]))) OR ("shortsight*"[All Fields] AND (y_1[Filter])) |  | in the last 1 year | ((("myopia"[MeSH Terms] OR "myopia"[All Fields] OR "myopias"[All Fields]) AND "2025/05/15 00:00":"3000/01/01 05:00"[Date - Publication]) OR ("nearsight*"[All Fields] AND "2025/05/15 00:00":"3000/01/01 05:00"[Date - Publication]) OR ("shortsight*"[All Fields] AND "2025/05/15 00:00":"3000/01/01 05:00"[Date - Publication])) AND (y_1[Filter]) | 2,898 | 01:56:47 | 2026/05/15 |
| **7** | "shortsight*"[All Fields] |  | in the last 1 year | ("shortsight*"[All Fields]) AND (y_1[Filter]) | 33 | 01:56:34 | 2026/05/15 |
| **6** | "nearsight*"[All Fields] |  | in the last 1 year | ("nearsight*"[All Fields]) AND (y_1[Filter]) | 30 | 01:56:21 | 2026/05/15 |
| **5** | "myopia"[MeSH Terms] OR "myopia"[All Fields] OR "myopias"[All Fields] |  | in the last 1 year | ("myopia"[MeSH Terms] OR "myopia"[All Fields] OR "myopias"[All Fields]) AND (y_1[Filter]) | 2,869 | 01:56:04 | 2026/05/15 |
| **4** | (("cost*"[All Fields] AND (y_1[Filter])) OR ("cost benefit analysis"[MeSH Terms] OR ("cost benefit"[All Fields] AND "analysis"[All Fields]) OR "cost benefit analysis"[All Fields] OR ("cost"[All Fields] AND "effectiveness"[All Fields]) OR "cost effectiveness"[All Fields] AND (y_1[Filter]))) OR ("cost benefit analysis"[MeSH Terms] OR ("cost benefit"[All Fields] AND "analysis"[All Fields]) OR "cost benefit analysis"[All Fields] OR ("economic"[All Fields] AND "evaluation"[All Fields]) OR "economic evaluation"[All Fields] AND (y_1[Filter])) |  | in the last 1 year | (("cost*"[All Fields] AND "2025/05/15 00:00":"3000/01/01 05:00"[Date - Publication]) OR (("cost benefit analysis"[MeSH Terms] OR ("cost benefit"[All Fields] AND "analysis"[All Fields]) OR "cost benefit analysis"[All Fields] OR ("cost"[All Fields] AND "effectiveness"[All Fields]) OR "cost effectiveness"[All Fields]) AND "2025/05/15 00:00":"3000/01/01 05:00"[Date - Publication]) OR (("cost benefit analysis"[MeSH Terms] OR ("cost benefit"[All Fields] AND "analysis"[All Fields]) OR "cost benefit analysis"[All Fields] OR ("economic"[All Fields] AND "evaluation"[All Fields]) OR "economic evaluation"[All Fields]) AND "2025/05/15 00:00":"3000/01/01 05:00"[Date - Publication])) AND (y_1[Filter]) | 100,699 | 01:55:48 | 2026/05/15 |
| **3** | "cost benefit analysis"[MeSH Terms] OR ("cost benefit"[All Fields] AND "analysis"[All Fields]) OR "cost benefit analysis"[All Fields] OR ("economic"[All Fields] AND "evaluation"[All Fields]) OR "economic evaluation"[All Fields] |  | in the last 1 year | ("cost benefit analysis"[MeSH Terms] OR ("cost benefit"[All Fields] AND "analysis"[All Fields]) OR "cost benefit analysis"[All Fields] OR ("economic"[All Fields] AND "evaluation"[All Fields]) OR "economic evaluation"[All Fields]) AND (y_1[Filter]) | 7,822 | 01:55:19 | 2026/05/15 |
| **2** | "cost benefit analysis"[MeSH Terms] OR ("cost benefit"[All Fields] AND "analysis"[All Fields]) OR "cost benefit analysis"[All Fields] OR ("cost"[All Fields] AND "effectiveness"[All Fields]) OR "cost effectiveness"[All Fields] |  | in the last 1 year | ("cost benefit analysis"[MeSH Terms] OR ("cost benefit"[All Fields] AND "analysis"[All Fields]) OR "cost benefit analysis"[All Fields] OR ("cost"[All Fields] AND "effectiveness"[All Fields]) OR "cost effectiveness"[All Fields]) AND (y_1[Filter]) | 14,835 | 01:54:55 | 2026/05/15 |
| **1** | "cost*"[All Fields] |  | in the last 1 year | ("cost*"[All Fields]) AND (y_1[Filter]) | 98,152 | 01:54:28 | 2026/05/15 |
