# Supplementary material for: Systematic review of atropine cost-effectiveness in childhood myopia management
Source: Front Pharmacol. 2026 Oct 16;17:1836579. doi: 10.3389/fphar.2026.1836579 (PMC13720285; doi:10.3389/fphar.2026.1836579)
Supplement: Supplementary file 4 [file Table4.docx]

Quality assessment of primary studies using ECOBIAS checklist

Table s4. Quality assessment of primary studies using ECOBIAS checklist

|  | **Bias** | **Questions considered in evaluating for the risk of bias, as per ECOBIAS checklist^1^** | **How the gradings were offered** | | | |
| --- | --- | --- | --- | --- | --- | --- |
| **PART A: Overall checklist for bias in economic evaluation** | | |  |  |  |  |
|  | | | **Hong et al 2022** | **Fricke et al 2023** | **Agyekum et al 2023b** | **Lee et al 2025** |
|  | Narrow perspective bias | - Was a societal perspective adopted? - If not, has a different perspective been justified? | Yes | Yes | Yes | Yes |
|  | Inefficient comparator bias | - Was the best alternative chosen as comparator? - Was current practice chosen as a comparator? Have all comparators been described in sufficient detail? | Yes Usual care with corrective lenses | Yes Full, single-vision correction | Yes  Single-vision lenses | Yes  Full, single-vision correction |
|  | Cost-measurement omission bias | - Were all costs relevant to the disease and intervention identified and considered? | Yes  Direct medical, monitoring, low vision care, productivity loss | Yes  Direct medical, productivity loss, lifetime complication costs | Yes  Direct and indirect costs including consultations, adverse events, and productivity loss | Yes  Direct medical, productivity loss, lifetime complication costs |
|  | Intermittent data collection bias | - Was the resource use measured continuously? | Unclear  the article does not provide enough information to determine whether the data was collected continuously or intermittently | Unclear  the article does not provide enough information to determine whether the data was collected continuously or intermittently | Unclear  the article does not provide enough information to determine whether the data was collected continuously or intermittently | Unclear  the article does not provide enough information to determine whether the data was collected continuously or intermittently |
|  | Invalid valuation bias | - Is the price calculation presented in a detailed manner? - Have reference prices been used? | Yes  NZ$2021 | Yes  USD2020 | Yes  USD2022 | Yes  USD2024 |

|  | Ordinal ICER bias | - Have cardinal scales for the outcomes measure in a CEA been used? | Yes  SER and Utility used in continuous value | Yes  SER | Yes  SER and AL | Yes  SER |
| --- | --- | --- | --- | --- | --- | --- |
|  | Double counting bias | - Are variables adequately checked for double counting? | Yes  Both screening and intervention cost are list separately | Yes Cost added up for each possible spherical equivalent level | Yes  Cost is described in detail | Yes  Cost by age group , and gender |
|  | Inappropriate discounting bias | - Have discounting rates from guidelines been applied? | Yes  3% and 6% | Yes 0%,3% and 5% | Yes  3% |  |
|  | Limited sensitivity analysis bias | - Have the four principles of uncertainty (methodological, structural, heterogeneity, parameter)   been considered in sufficient detail? | Yes | Yes | Yes |  |
|  | Sponsor bias | - Have sponsorships been disclosed? - Is the study protocol freely accessible? | Sponsorship disclosed  Protocol not available | Sponsorship disclosed  Protocol not available | Sponsorship disclosed  Protocol not available | Sponsorship disclosed  Protocol not available |
|  | Reporting and dissemination bias | - Has the study/trial been listed in a trial register? - Have all results been reported according to the study protocol? | Unclear | Unclear  Treatment protocol where mention and illustrated in the paper | Unclear | Unclear |
| **PART B: Model-specific aspects of bias in economic evaluation** | | |  |  |  |  |
|  | | |  |  |  |  |
|  | Structural assumption bias | - Is the model structure in line with coherent theory? - Do treatment pathways reflect the nature of disease? | Yes  Normal, myopia to high myopia, pathological and blindness | Unclear  Custom protocol with dynamic disease progression | Yes  Low, Moderate, High | Unclear  Custom protocol with dynamic disease progression |
|  | No treatment comparator bias | - Is there an adequate comparator, i.e. care as usual? | Yes | Yes | Yes | Yes |
|  | Wrong model bias | - Is the model chosen adequate regarding the decision problem? | Yes  Decision tree with Markov model | Unclear  Custom model that follows the disease development stage | Yes  Markov model | Unclear  Custom model that follows the disease development stage |
|  | Limited time  horizon bias | - Was a lifetime horizon chosen? - Were shorter time horizons adequately justified? | Lifetime  Justified up to 80 years (New Zealand live expectancy) | Lifetime | 5-years based on current RCT length | Lifetime |

|  | Bias related to data identification | - Are the methods of data identification transparent? - Are all choices justified adequately? - Do the input parameters come from high quality and well-designed studies? | Yes  Prevalence data from Australia  Efficacy data from RCT ATOM2  Cost data from online Reserve bank of New Zealand | Yes  Data from China and Australia | Yes  Data from Hong Kong | Yes |
| --- | --- | --- | --- | --- | --- | --- |
|  | Bias related to baseline data | - Are probabilities, for example, based on natural history data? - Is transformation of rates into transition probabilities done accurately? | Data were validated and model were calibrated | Myopia progressions were modelled from China and adjusted to Australian child using method of Donovan et al | Yes | Progression model from China Han (Tim et al) as for fast progressor, data from informant |
|  | Bias related to treatment effects | - Are relative treatment effects synthesized using appropriate metanalytic techniques? - Are extrapolations documented and well justified? - Are alternative assumptions explored regarding extrapolation? | Yes | Yes | Yes | Yes |
|  | Bias related to quality-of-life weights (utilities) | - Are the utilities incorporated appropriate for the specific decision problem? | Yes | No utility reported | No utility reported | No utility reported |
|  | No transparent data incorporation bias | - Is the process of data incorporation transparent? - Are all data and their sources described in detail? | Yes | Yes | Yes | Yes |
|  | Limited scope bias | - Have the four principles of uncertainty (methodological, structural, heterogeneity, parameter) been considered? | Yes | Yes | Yes | Yes |
|  | Bias related to internal  consistency. | - Has internal consistency in terms of mathematical logic been evaluated? | Yes – source and rational is included | Yes | Yes | Yes |
